# Brain Amyloid Deposition and Longitudinal Cognitive Decline in Nondemented Older Subjects: Results from a Multi-Ethnic Population

**DOI:** 10.1371/journal.pone.0123743

**Published:** 2015-07-29

**Authors:** Yian Gu, Qolamreza R. Razlighi, Laura B. Zahodne, Sarah C. Janicki, Masanori Ichise, Jennifer J. Manly, D. P. Devanand, Adam M. Brickman, Nicole Schupf, Richard Mayeux, Yaakov Stern

**Affiliations:** 1 The Taub Institute for Research in Alzheimer’s Disease and the Aging Brain, Columbia University, New York, New York, United States of America; 2 The Department of Neurology, Columbia University, New York, New York, United States of America; 3 The Gertrude H. Sergievsky Center, Columbia University, New York, New York, United States of America; 4 Department of Radiology, Columbia University Medical College, New York, New York, United States of America; 5 Department of Psychiatry, Columbia University College of Physicians and Surgeons, New York, New York, United States of America; 6 The Division of Epidemiology, Joseph P. Mailman School of Public Health, Columbia University, New York, New York, United States of America; Johns Hopkins School of Medicine, UNITED STATES

## Abstract

**Objective:**

We aimed to whether the abnormally high amyloid-β (Aβ) level in the brain among apparently healthy elders is related with subtle cognitive deficits and/or accelerated cognitive decline.

**Methods:**

A total of 116 dementia-free participants (mean age 84.5 years) of the Washington Heights Inwood Columbia Aging Project completed 18F-Florbetaben PET imaging. Positive or negative cerebral Aβ deposition was assessed visually. Quantitative cerebral Aβ burden was calculated as the standardized uptake value ratio in pre-established regions of interest using cerebellar cortex as the reference region. Cognition was determined using a neuropsychological battery and selected tests scores were combined into four composite scores (memory, language, executive/speed, and visuospatial) using exploratory factor analysis. We examined the relationship between cerebral Aβ level and longitudinal cognition change up to 20 years before the PET scan using latent growth curve models, controlling for age, education, ethnicity, and Apolipoprotein E (APOE) genotype.

**Results:**

Positive reading of Aβ was found in 41 of 116 (35%) individuals. Cognitive scores at scan time was not related with Aβ. All cognitive scores declined over time. Aβ positive reading (B = -0.034, p = 0.02) and higher Aβ burden in temporal region (B = -0.080, p = 0.02) were associated with faster decline in executive/speed. Stratified analyses showed that higher Aβ deposition was associated with faster longitudinal declines in mean cognition, language, and executive/speed in African-Americans or in APOE ε4 carriers, and with faster memory decline in APOE ε4 carriers. The associations remained significant after excluding mild cognitive impairment participants.

**Conclusions:**

High Aβ deposition in healthy elders was associated with decline in executive/speed in the decade before neuroimaging, and the association was observed primarily in African-Americans and APOE ε4 carriers. Our results suggest that measuring cerebral Aβ may give us important insights into the cognitive profile in the years prior to the scan in cognitively normal elders.

## Introduction

A hallmark of Alzheimer’s disease (AD), the leading cause of dementia in the elderly, is the deposit of amyloid-β (Aβ) in the brain. However, postmortem studies have found approximately 30% of cognitively normal elderly also show Aβ deposition in the brain [1–3]. Similar to pathological data, a 20%~30% prevalence of Aβ deposition in brain has been seen among cognitively normal, asymptomatic elderly using in vivo positron emission tomography (PET) imaging of radioligands that bind to fibrillar Aβ in amyloid plaques[4–7].

It has been hypothesized that Aβ deposition in the brain is an early event in the pathogenesis of AD [8], and that clinically normal individuals with Aβ deposits might be in a preclinical, prodromal stage of AD [9]. Supporting this hypothesis, several prospective studies [10–13] found that healthy older adults with higher cerebral Aβ had a faster cognitive decline following PET imaging than those with lower cerebral Aβ during 18-month follow up. However, other studies have reported that cognitively healthy older adults with high cerebral Aβ were not different from those with low cerebral Aβ on the rate of cognitive change over 24 months[14,15]. In addition, cross-sectional studies [16] have also yielded inconsistent results, with some studies finding that Aβ positive healthy individuals have worse cognitive performance[7,17–19] and others reporting no association [4,6,20–24]. Thus, it remains unclear whether the abnormally high Aβ level in the brain among apparently healthy elderly people indicates an underlying subtle cognitive deficit and/or accelerated cognitive decline.

As currently prospective amyloid PET data do not have long duration of follow-up, examining cognitive trajectory before PET imaging is a useful way to help understand the implications of cerebral Aβ deposition on cognition among non-demented subjects. Several retrospective longitudinal studies [25–29] have consistently found among apparently normal elders that, compared to individuals with Aβ negative or lower levels of Aβ, individuals with positive or higher levels of Aβ had faster cognitive decline over a period of time prior to scanning. While the findings from these retrospective longitudinal studies seem to be quite consistent, most of the studies included predominantly a single ethnic group of European origin[25–29]. Little is known about whether cerebral Aβ is associated different patterns of cognitive change over time among other ethnic groups such as African-Americans. In addition, except for one study[29], previous studies have primarily included non-demented younger-old participants who were 65–80 years old[25–28]. Since AD is highly age-related[30], it is also important to know whether there is similar, or higher, prevalence of cerebral Aβ deposition in non-demented older-old individuals and whether such deposition has similar implications regarding the cognitive change in the preceding years.

In this study, we evaluated the prevalence and level of Aβ deposition using ^18^F-Florbetaben in a multi-ethnic elderly population with an average age of nearly 85 years, and examined whether individuals with higher brain level of Aβ deposition had faster rate of cognitive decline than those with lower levels of brain Aβ deposition in the decade prior to scanning.

## Methods

### Study Participants

Subjects were selected from those participating in the Washington Heights Inwood Columbia aging project (WHICAP). The WHICAP participants were identified from a probability sample of Medicare beneficiaries aged 65 or older, residing in northern Manhattan[31]. The initial sample for this study included 2,776 participants of the ongoing WHICAP II cohort. Briefly, at entry, trained examiners obtained each participant’s demographic information, medical and neurological history, and conducted a standardized physical and neurological examination. Participants were followed at intervals of approximately 1.5 years, repeating all the evaluations. Consensus diagnoses were made by a team of neuropsychologists and neurologists based on standard research criteria[32]. The diagnosis of mild cognitive impairment (MCI) in this cohort has been described elsewhere[33] and was based on Petersen [34] criteria.

Since 2004, we systematically collected high-resolution magnetic resonance imaging (MRI) data on 769 dementia-free WHICAP II participants. Detailed description of the neuroimaging subsample can be found in our previous report[35]. In 2009, we began to measure brain Aβ burden using a PET tracer with the goal of imaging 728 participants who were free of dementia at their previous visit. The subjects who participated so far in the ongoing PET study (n = 125) were younger at the time of their first magnetic resonance imaging (MRI) scan (mean age 79.2 vs. 80.3 years, p = 0.01), had more years of education (12.4 vs. 10.4 years, p = 0.0001), and were less likely to be Hispanics (21% vs 39%; p<0.0001) than those without PET scans (n = 603). Those with and without PET scan were not different in terms of their gender, apolipoprotein ε4 (APOE) status, or comorbidities (hypertension, diabetes, or heart disease). A total of 9 participants who were diagnosed with dementia around the time of the PET imaging were further excluded from the analysis. Thus, the current analysis included 116 dementia-free participants. The subjects had been followed up for an average of 11.8 years (range 3.2 to 20.4 years) with 5.68 visits (2 to 11 visits) prior to the PET scan.

The Columbia University Institutional Review Board has reviewed and approved this project. All individuals provided written informed consent.

### Cognitive evaluation

Cognition was determined using a neuropsychological battery [36] which was administered either in English or Spanish at baseline and each follow-up visit. Selected neuropsychological tests scores were combined into four composite scores (memory, language, executive/speed, and visuospatial) based on an exploratory factor analysis using principal axis factoring and oblique rotation[36]. Memory was assessed with the Selective Reminding Test [37], including total recall, delayed recall, and delayed recognition, and with recognition from the Benton Visual Retention Test[38]. The language domain included measures of naming, letter fluency, category fluency[39], verbal abstract reasoning[40], and repetition and comprehension[41]. Executive-Speed was assessed with the Color Trails test1 and 2 [42], and the times taken to complete the tasks were used as the dependent measures. Visuospatial ability was assessed with the Rosen Drawing Test[43], the BVRT–Matching[38], and the Identities and Oddities subtest of the Mattis Dementia Rating Scale[44].

Means and standard deviations (SD) were calculated from baseline scores for non-demented WHICAP subjects controlling for age, race/ethnicity, and years of education. Z-scores for each of the cognitive measures were calculated and then averaged to create a composite Z-score for each of the four domains. These factor domain scores were subsequently averaged to produce a composite “mean cognition” z-score. A higher z-score indicates better cognitive performance.

### Image Acquisition, Processing, and Analysis

#### ^18^F-Florbetaben

All image processing and analyses were conducted by persons blinded to the clinical status and cognitive test results of participants. Participant preparation consisted of intravenous catheterization followed by the bolus injection (over 10–20 sec) of 10 mCi of ^18^F-Florbetaben. The PET scans were acquired over a period of 20 minutes in 4×5 minute frames on an MCT PET/CT scanner (Siemens) in dynamic, 3D imaging mode beginning 50 min after injection of ^18^F- Florbetaben. Transmission scans were done prior to the scan. An accompanying structural CT scan (in-plane resolution = 0.58×0.58 mm, slice thickness = 3mm, field of view = 29.6×29.6 cm^2^, number of slices = 75) was also acquired in the same machine at the same time as the PET scan.

#### Visual rating

We used a method similar to that of Barthel and colleagues [45] for the visual classification of brain Aβ deposition. This approach has also been used in the blinded reads of phase 3 trials [46]. The visual assessment was based on the PET scans alone without co-registration of MRI brain scans. Florbetaben binding in the specific regions [frontal cortex (FRC); temporal cortex (TMP); parietal cortex (PAR); cingulate gyrus (CG); and occipital cortex] were rated as visual Aβ (vAβ) positive if the activity was greater than that in the adjacent white matter, otherwise vAβ negative. The subject received a positive Aβ reading if any of the regions was considered as positive. Two readers (SJ and MI) worked independently, blind to all clinical data, cognitive test results, and the quantitative Aβ measures (see below) of the participants. After the independent reads, discordant cases (17%) were reviewed by the two readers together to reach a consensus. The overall Kappa was 0.61, suggesting a fair to good agreement between the readers[47].

#### Quantitative image analysis

Each participant received a brain MRI using a 1.5T Philips Intera scanner (TR/TE 20/2.1 ms/ Flip angle 20 deg/ 256 x 256 matrix / acquisition time 8’ 09”/ 1.3 mm slice thickness/ 105 slices). FreeSurfer (http://surfer.nmr.mgh.harvard.edu/), the MRI software package comprising a suite of automated tools for segmentation, reconstruction, and derivation of regional volumes and surface-based rendering, was used for derivation of regions-of-interest (ROI). In total, 95 ROIs masks (35x2 cortical, 23 subcortical, and cerebellar gray matter and white matter) were extracted from the structural T1 image. Four set of non-overlapping ROIs were selected: FRC; TMP; PAR; and CG for the statistical analyses.

**Fig 1:**
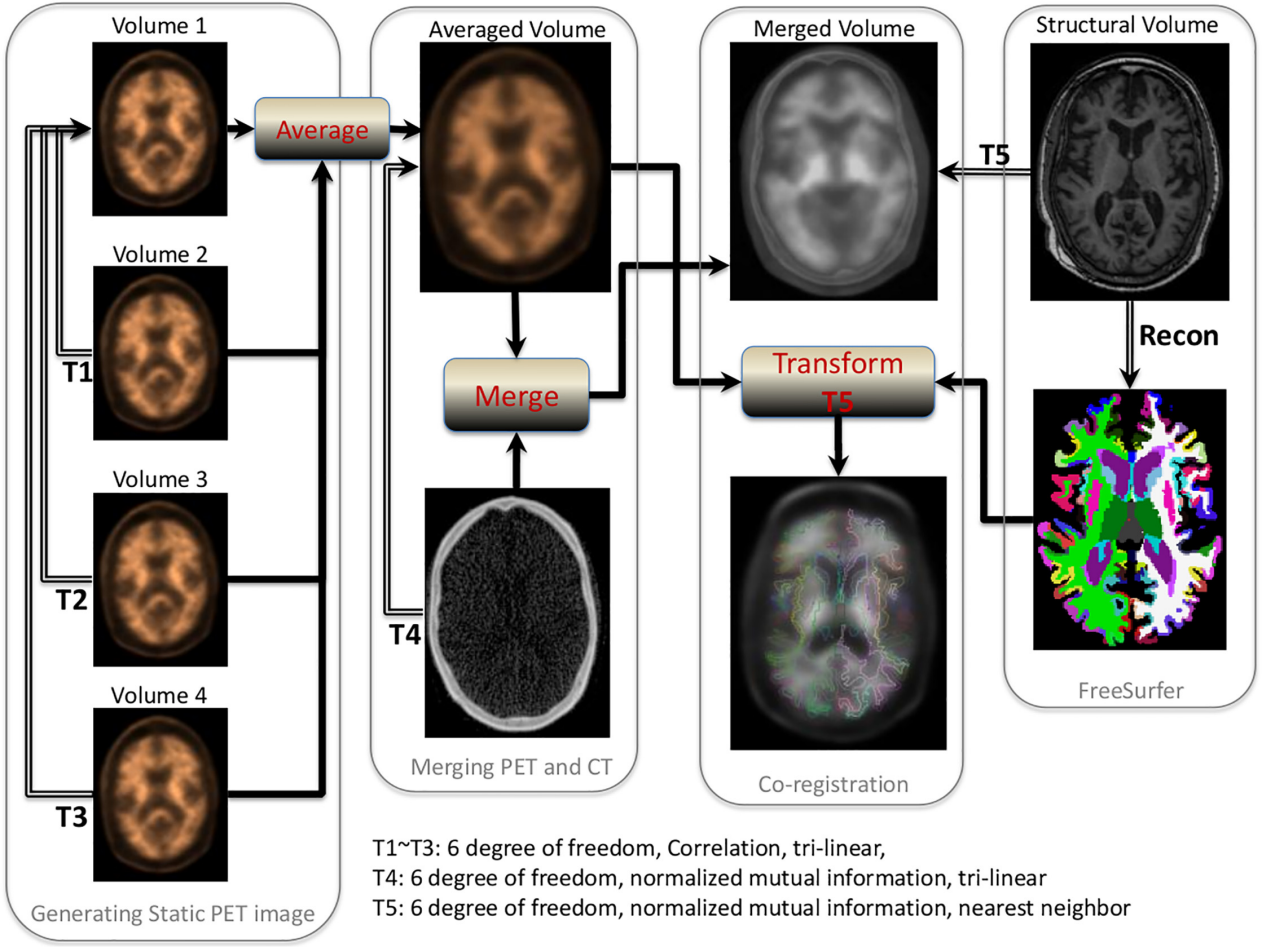
Procedures for quantitative PET amyloid analysis.

Dynamic PET frames (4 scans) were aligned to the first frame using rigid-body registration and a static PET image was obtained by averaging the four registered frames. The static PET image was registered with the CT to obtain the transformation matrix, and the inverse of this transformation matrix then transferred the CT image to static PET image space. The CT and static PET image were merged to generate a composite image in the PET static space. Each individual’s structural T1 image in FreeSurfer space was also registered to the participant’s merged image using normalized mutual information and tri-linear interpolation to obtain the second transformation matrix. A combination of the two transformation matrices was used to transfer the 4 regional masks and the cerebellar gray matter from FreeSurfer space to static PET image space using nearest neighbor interpolation. These 4 regional masks in static PET space were used to extract the regional PET data. The procedures are summarized in Fig 1.

The standardized uptake value, defined as the decay-corrected brain radioactivity concentration normalized for injected dose and body weight, was calculated at selected regions. The standardized uptake value was then normalized to cerebellum to derive the standardized uptake value ratio (SUVR), which was the measurement used in the analyses. Analyses incorporated both the individual ROIs (including TMP, PAR, CG, and FRC) and an overall mean value of amyloid burden across the ROIs. The T1 scan was not available for 11 subjects so SUVR could not be calculated and we included the remaining 105 subjects in the analysis involving SUVR.

### Covariates

Information about birthdate, sex, education, and ethnicity was obtained from baseline interviews. Age (years) at time of scan was calculated and used as a continuous variable. Education (years) was used as a continuous variable. Ethnic group was based on self-report using the format of the 2000 U.S. census. Participants were then assigned to one of four groups: African American (non-Hispanic), Hispanic, White (non-Hispanic) or Other. Two dummy variables were created to indicate the three major ethnic groups (White, African-American, and Hispanic, with White as the reference group). Sex was used as a dichotomous variable with male as the reference. APOE ε4 genotype was treated as a dichotomous variable: absence (as reference) versus presence of either 1 or 2 ε4 alleles.

### Statistical Analysis

The cross-sectional associations between Aβ SUVR values and cognitive scores at the time of scan acquisition were examined using multivariable linear regression models, adjusted for age, gender, education, ethnicity, and APOE ε4 genotype.

We used latent growth curve models [48] to test whether the rate of cognitive decline in neuropsychological test scores varied according to Aβ status (positive or negative by visual reading, quantitative SUVR level). We modeled cognitive trajectories over these 5 visits leading up to the PET scan. Time was parameterized as years since the initial visit. Models were initially unadjusted, and then adjusted for age, sex, education, ethnicity, and APOE genotype. As we were particularly interested in whether the PET Aβ level-associated difference of cognitive trajectories varied by gender, ethnic groups, and APOE genotype, we decided a priori to perform stratified analysis by subgroups of gender, ethnicity, and APOE genotype.

MCI is often a prodromal stage of AD. Thus subjects having MCI might be different than the cognitively normal subjects in terms of their clinical, cognitive, and brain pathological status, as well as the relationship among these factors. To examine the relationship between PET Aβ and cognitive change among cognitively healthy aging subjects only, we performed sensitivity analysis by excluding participants who were diagnosed with MCI at the time of PET scan.

Statistical analyses were performed in SPSS (version 18) and M-plus version 7. All p-values were based on two-sided tests with significance level set at 0.05.

## Results

### Demographic/clinical characteristics and PET Aβ

Forty-one (35%) subjects were classified as vAβ positive (Table 1). Participants had a mean global SUVR of 1.27 (SD = 0.22) (Table 1). Participants who had positive vAβ had higher Aβ SUVR values globally and in each of the ROIs (Table 1). Global Aβ SUVR and Aβ SUVR in the ROIs were all highly correlated (correlation coefficients >0.9 and p<0.0001 for all).

**Table 1:** Characteristics of study participants according to negative or positive visual reading of brain Aβ imaging.

	Total	Negative	Positive	*p*
N (%)	116	75 (65%)	41 (35%)	/
Follow up time(years), mean (SD)	11.8 (2.9)	11.6 (2.8)	12.2 (3.0)	0.30
**Age (years), mean (SD)**	**84.5 (4.6)**	**83.8 (4.4)**	**85.9 (4.7)**	**0.02**
Education (years), mean (SD)	12.71 (3.9)	12.61 (3.52)	12.76 (4.1)	0.85
Race/Ethnicity, N(%)				0.46
White	40 (35)	24 (32)	16 (39)	
African-Americans	53 (46)	36 (48)	17 (42)	
Hispanic	22 (19)	15 (20)	7 (17)	
Other	1 (1)	0	1 (2)	
Female, N(%)	74 (64)	45 (60)	29 (71)	0.25
**APOE ε4 status, N(%)**				**0.015**
**0 ε4 allele**	**79 (68)**	**58 (77)**	**21 (51)**	
**1 ε4 allele**	**33 (28)**	**15 (20)**	**18 (44)**	
**2 ε4 alleles**	**4 (3.4)**	**2 (2.7)**	**2 (4.9)**	
**APOE ε4 1 or 2 alleles, N(%)**	**37 (31)**	**17 (23)**	**20 (49)**	**0.004**
MCI, N(%)	17 (15)	10 (14)	7 (18)	0.55
Mean Cognition Z-score, mean (SD)	0.38 (0.48)	0.35 (0.40)	0.40 (0.52)	0.55
Memory Z-score, mean (SD)	0.19 (0.68)	0.27 (0.68)	0.04 (0.68)	0.08
Language Z-score, mean (SD)	0.53 (0.55)	0.52 (0.57)	0.55 (0.51)	0.78
Visuospatial Z-score, mean (SD)	0.45 (0.44)	0.42 (0.50)	0.49 (0.29)	0.40
Speed Z-score, mean (SD)	0.41 (0.83)	0.46 (0.87)	0.32 (0.74)	0.41
**Global SUVR** ^**┼**^ **, mean (SD)**	**1.27 (0.22)**	**1.17 (0.14)**	**1.48 (0.23)**	**<0.0001**
**FRC SUVR** ^**┼**^ **, mean (SD)**	**1.25 (0.24)**	**1.14 (0.15)**	**1.47 (0.25)**	**<0.0001**
**TMP SUVR** ^**┼**^ **, mean (SD)**	**1.19 (0.21)**	**1.10 (0.13)**	**1.38 (0.24)**	**<0.0001**
**PAR SUVR** ^**┼**^ **, mean (SD)**	**1.18 (0.23)**	**1.08 (0.14)**	**1.39 (0.24)**	**<0.0001**
**CG SUVR** ^**┼**^ **, mean (SD)**	**1.45 (0.24)**	**1.35 (0.16)**	**1.67 (0.24)**	**<0.0001**

^┼^ Limit to 105 subjects who had both clinical reading (72 negative and 33 positive readings) and quantitative data.

Participants who had positive vAβ were older and were more likely to carry at least one APOE ε4 allele, compared with those with negative vAβ (Table 1).

SUVR values tended to increase with increasing age, although not significantly (Pearson’s correlation coefficients of age with global, FRC, TMP, PAR, and CG were r = 0.16, p = 0.11; r = 0.15, p = 0.14; r = 0.13, p = 0.18; r = 0.18, p = 0.07; r = 0.16, p = 0.10, respectively.). Participants with one or two APOE ε4 alleles had significantly higher SUVR than those without ε4 allele globally and in each region (Table A in S1 File). Women tended to have higher SUVR than men globally and in all regions except for FRC (Table A in S1 File). For both males and females, those who had positive vAβ had higher Aβ SUVR (Table B in S1 File).

### Cross-sectional analysis

The cognitive scores did not differ between participants with positive and negative vAβ (Table 1) and were not correlated with any of the Aβ SUVR values (correlation coefficients were among the range of -0.1 to 0.1, and were not significant). Multivariable regression analysis adjusted for age at scan, sex, education, ethnicity, and APOE also showed that there was no association between any of the cognitive scores and PET Aβ (Table C in S1 File).

### Longitudinal analysis

All cognitive z-scores declined significantly over time during the follow-up period before imaging (all unadjusted p<0.0001 except for visuospatial which had p = 0.014). Subjects with positive vAβ declined in executive/speed at a rate that was 0.034 points/year greater than that of subjects with negative vAβ (Table 2). Higher Aβ burden in the temporal region were associated with faster decline in speed (one unit increase in SUVR values was associated with 0.080 points/year faster decline) (Table 2). PET Aβ was not associated with decline rate of other cognitive scores. Additionally adjusting for MCI status did not change the results materially (Table 2).

**Table 2:** Brain Aβ in relation to the rate of cognitive decline during the decade prior to PET scan among non-demented participants.

		Visual rating		Global		FRC		TMP		PAR		CG	
		B	p	B	p	B	p	B	p	B	p	B	p
Mean Cog.	Model 1	-0.011	0.08	-0.015	0.33	-0.014	0.36	-0.022	0.17	-0.011	0.49	-0.011	0.43
	Model 2	-0.01	0.10	-0.018	0.25	-0.014	0.33	-0.025	0.09	-0.015	0.31	-0.012	0.39
	Model 3	-0.011	0.10	-0.026	0.06	-0.021	0.13	**-0.032**	**0.02**	-0.023	0.09	-0.021	0.09
Memory	Model 1	-0.002	0.08	0.002	0.95	0.008	0.77	-0.01	0.75	0.001	0.98	0.007	0.80
	Model 2	-0.017	0.10	-0.001	0.96	0.007	0.78	-0.014	0.62	-0.006	0.82	0.006	0.82
	Model 3	-0.01	0.36	0.005	0.87	0.014	0.62	-0.013	0.67	0.001	0.98	0.014	0.60
Language	Model 1	-0.003	0.62	-0.008	0.60	-0.002	0.89	-0.01	0.52	-0.013	0.36	-0.005	0.72
	Model 2	-0.002	0.70	-0.009	0.54	-0.002	0.88	-0.012	0.44	-0.015	0.27	-0.005	0.70
	Model 3	-0.001	0.84	-0.018	0.21	-0.009	0.54	-0.023	0.10	-0.023	0.08	-0.024	0.91
Visuo-spatial	Model 1	0.005	0.41	-0.009	0.60	-0.008	0.60	-0.008	0.65	-0.004	0.78	-0.012	0.45
	Model 2	0.005	0.39	-0.01	0.57	-0.009	0.59	-0.009	0.60	-0.006	0.72	-0.013	0.43
	Model 3	0.003	0.68	-0.009	0.62	-0.005	0.78	-0.01	0.60	-0.009	0.63	-0.011	0.50
Speed	Model 1	**-0.034**	**0.02**	-0.057	0.11	-0.057	0.10	**-0.08**	**0.02**	-0.037	0.25	-0.039	0.26
	Model 2	**-0.033**	**0.02**	-0.064	0.07	-0.061	0.07	**-0.088**	**0.01**	-0.046	0.16	-0.044	0.20
	Model 3	**-0.039**	**0.01**	**-0.072**	**0.03**	**-0.068**	**0.03**	**-0.091**	**0.00**	-0.055	0.07	-0.056	0.08

Results from latent growth curve models. B weights were the estimates for the association between Aβ and cognitive change. A positive B indicated that having higher level of Aβ deposition (or positive compared to negative vAβ) was associated with less annual decline in cognitive scores, while a negative B indicated faster decline. Model 1: All subjects, adjusted for age at PET scan, gender, ethnicity, education, APOE ε4 genotype. Model 2: All subjects, adjusted for above covariates and MCI status. Model 3: Sensitivity analysis: Healthy aging subjects only (excluding 17 MCI subjects), adjusted for age at PET scan, gender, ethnicity, education, APOE ε4 genotype.

### Stratified analyses by APOE ε4 genotype, ethnic groups, or gender

Stratified analysis showed that vAβ positivity, higher level of global Aβ deposition or Aβ deposition in each of the four ROIs (data not shown), was associated with a larger amount of annual decline on mean cognition, language, and executive/speed scores in African-Americans but not in Whites (Table 3), and in APOE ε4 carriers but not in APOE ε4 negative subjects (Table 3). Aβ deposition was also related with a faster decline in memory in APOE ε4 carriers, but not in APOE ε4 negative subjects (Table 3). The sample size of Hispanics was too small to yield trustworthy parameter estimation from the latent growth curve models. We found positive vAβ was associated with faster decline in mean cognition in males only. The vAβ and global Aβ SUVR were not associated with other cognitive score decline rate in either males or females (Table 3).

**Table 3:** Brain Aβ in relation to the rate of cognitive decline during the decade prior to PET scan among non-demented participants, stratified by APOE ε4 genotype, ethnicity, and gender.

	Visual Aβ rating Positive vs. Negative				Global Aβ			
	APOE ε4+		APOE ε4 -		APOE ε4+		APOE ε4 -	
	B	*p*	B	*p*	B	*p*	B	*p*
Mean Cog.	**-0.028**	***0*.*004***	-0.002	*0*.*805*	**-0.04**	***0*.*019***	0.004	*0*.*802*
Memory	**-0.048**	***0*.*003***	-0.004	*0*.*795*	-0.064	*0*.*124*	0.058	*0*.*118*
Language	**-0.026**	***0*.*006***	0.009	*0*.*166*	**-0.042**	***0*.*006***	NA	*NA*
Visuospatial	0.133	*0*.*125*	0.116	*0*.*247*	0.293	*0*.*564*	-0.073	*0*.*802*
Speed	-0.025	*0*.*086*	-0.025	*0*.*175*	**-0.057**	***0*.*043***	-0.051	*0*.*293*
	White		African-Americans		White		African-Americans	
	B	*p*	B	*p*	B	*p*	B	*p*
Mean Cog.	-0.005	*0*.*63*	**-0.023**	***0*.*002***	0.022	*0*.*39*	**-0.056**	***<0*.*0001***
Memory	-0.002	*0*.*33*	-0.027	*0*.*072*	0.039	*0*.*39*	-0.02	*0*.*60*
Language	0.004	*0*.*67*	**-0.019**	***0*.*014***	0.015	*0*.*51*	**-0.048**	***0*.*001***
Visuospatial	0.008	*0*.*36*	0.002	*0*.*85*	0.011	*0*.*77*	-0.023	*0*.*39*
Speed	*-0*.*013*	*0*.*52*	**-0.047**	***0*.*017***	-0.013	*0*.*83*	**-0.115**	***0*.*001***
	Males		Females		Males		Females	
	B	*p*	B	*p*	B	p	B	*p*
Mean Cog.	**-0.018**	***0*.*04***	-0.008	*0*.*34*	-0.009	*0*.*74*	-0.017	*0*.*30*
Memory	-0.026	*0*.*12*	-0.019	*0*.*16*	0.012	*0*.*74*	-0.014	*0*.*71*
Language	-0.022	*0*.*69*	0.008	*0*.*28*	NA	*NA*	-0.001	*0*.*95*
Visuospatial	-0.006	*0*.*44*	0.009	*0*.*29*	-0.022	*0*.*37*	-0.002	*0*.*91*
Speed	-0.018	*0*.*35*	-0.035	*0*.*07*	-0.059	*0*.*29*	-0.051	*0*.*15*

Results from latent growth curve models, adjusted for age at PET scan, sex, gender, ethnicity, education, and APOE ε4 genotype, except for the variable in stratification. B weights were the estimates for the association between Aβ and cognitive change. A positive B indicated that having higher level of Aβ deposition (or positive compared to negative vAβ) was associated with less annual decline in cognitive scores, while a negative B indicated faster decline. NA: trustworthy parameter estimation was Not Available.

### Sensitivity analysis

We compared the demographic, clinical, cognitive, and brain pathological profiles of MCI with that of cognitively normal participants (Table D in S1 File). As expected, the MCI subjects in general started with significantly lower cognitive performance than the non-MCI subjects, and their cognitive scores were also much lower than non-MCI subjects at the time of the scan visit. There were no difference of demographic, genetic, and Aβ status between MCI and non-MCI subjects, except that no Hispanics had MCI while 14.5% of Whites and 15.1% of African-Americans had MCI.

After excluding 17 MCI participants from the analysis, we found the results remained similar to the main analysis, although the associations were slightly stronger compared to the results when MCI subjects were included (Table 2).

## Discussion

In this multiethnic, non-demented elderly population, we found participants with higher load of Aβ depositions experienced an accelerated decline in executive/speed in the decade prior to the scan. Furthermore, we found the association between Aβ deposition and cognitive trajectory only among African-Americans or among APOE ε4 positive subjects.

Approximately 35% of the study participants had positive Aβ depositions according to visual reading of the PET scans, a proportion similar to other reports of Aβ deposition in healthy elderly based on either imaging techniques or postmortem pathological analysis [1–7]. Besides being slightly older, these vAβ positive subjects were more likely to have APOE ε4 allele than those with negative visual Aβ readings. These findings are consistent with previous reports[25–29]. We found the average retention ratio of Aβ in the four ROIs compared to cerebellum were 1.27, similar to what has been reported in other populations using florbetaben[24,45] or PiB[27,28].

A recent meta-analysis revealed mixed evidence for cross-sectional association between cognitive function and Aβ deposition, although small effects on episodic memory or global cognition were found according to amyloid burden[16]. In our cross-sectional analysis, Aβ burden in general was not associated with concurrent cognitive scores. This null association has also been reported by previous studies either using florbetaben [24] or PiB Aβ[4,21] as the PET tracer. While it is possible that amyloid status in healthy elderly provides no direct link with the cognitive profile, there are other potential reasons. For example, although in normal older individuals Aβ deposition may be the earliest pathological event before clinical decline, tau or other pathophysiologic processes such as brain atrophy may also be involved[28]. Thus, cognitive variation may be associated with the combined effects of all these physiopathological indicators but not a single one of them.

We found that Aβ burden was associated with more rapid decline in executive/speed in the years prior to Aβ imaging in an older population with an average age of 85. This finding is consistent with previous reports of higher PET Aβ being associated with greater decline in executive functions[27,29]. Previous studies have also found higher Aβ burden was associated with steeper trajectories of verbal memory [27], visual memory[29], semantic fluency[29], working memory[26], and visuospatial ability[26] in non-demented elderly. An early study[25] found clinically defined cognitively ‘declining’ subjects were much more likely to show cortical PiB binding than ‘stable’ subjects. Landau and colleagues found that subjects with positive florbetapir declined significantly faster than those with negative florbetapir on Cognitive subscale of the Alzheimer’s Disease Assessment Scale [28]. Thus, our results add to the existing body of evidence that Aβ deposition in the brain might be associated with preceding cognitive trajectory. Nevertheless, results were not always consistent. For example, association of Aβ with visual memory was found in one[29] but in another other study[27]. Furthermore, the use of different measures of cognition and different tracers precludes a direct comparison of the findings across studies. With regard to regional Aβ deposition, we found significant associations between decline in executive/speed and Aβ deposition in the temporal region, a region that was also reported in a previous study[27]. The Aβ deposition in the frontal region, the region that is involved for executive/speed function[49], was also associated with executive/speed among cognitively normal subjects.

In the sensitivity analysis by excluding subjects who were considered as MCI at the time of scan visit, we found positive vAβ, higher Aβ SUVR in global, FRC, and TMP regions were associated with faster decline in executive/speed score, and SUVR values in the TMP region was associated with faster decline in mean cognition. The associations seemed to be even slightly stronger compared to the results from the entire study population. The exact reason is unknown. It may not simply be due to the lower starting cognition score positioning the MCI subjects less room to deteriorate, as MCI subjects continued to decline over time. Other potential explanation could be that, Aβ presence triggers the cascade of cognitive decline in cognitively healthy subjects, while for subjects who developed MCI, the initial cognitive decline has already happened and the continued decline depends less on Aβ burden but more on other pathological changes such as Tau or structural brain changes[50]. Nevertheless, these hypotheses need to be tested in future studies.

Ethnic differences in the associations between Aβ and prior cognitive change have not been previously reported, but might be important considering the increasingly diverse general population in the US. We found higher Aβ deposition was associated with faster decline in language, speed, and mean cognitive scores among African-Americans only. It remains unknown whether the findings were contributed by factors other than biological interaction, such as the smaller sample size of Whites than African-Americans, and the slightly lower percentage of women and APOE ε4 carriers in Whites. We also found that higher Aβ deposition was associated with a faster decline in cognitive scores only in APOE ε4 carriers. This observation is in line with cross-sectional evidence[18,19], and is probably not surprising as APOE ε4 constitutes the main genetic risk factor for AD[51] and is supposed to be involved in the formation and clearance of Aβ[52,53]. However, it remains to be confirmed in future studies. We found no major difference of the association between Aβ deposition and cognition between females and males.

Some limitations of the current study need to be noted. Our study did not examine whether brain Aβ deposit is associated with future cognitive change. However, prospective follow-up of these participants is ongoing, and future data on cognitive assessments will assist us in understanding the relationship between brain Aβ and subsequent cognitive change. Secondly, we had a smaller percent of Hispanic participants in the current study sample than the overall WHICAP population, and due to the small number of Hispanic subjects we were not able to estimate the association between Aβ and cognitive change. More Hispanic participants will be recruited into the imaging study in the future in order to extrapolate the results to the source community population. The interreader agreement for vAβ was not perfect and might be lower than some other studies[54–56]. However, the potential misclassification might have biased the results toward an inflated type II error rather than a false positive result (type I error). Thus, despite imperfect vAβ agreement, our confidence remains with regard to the significant association between vAβ and cognitive decline.

Our study has many strengths. While most of the previous studies examining cognition and PET Aβ included predominantly a single race/ethnicity group (mainly Whites), our study included an ethnically diverse community-based population. Furthermore, separate estimates of the association between Aβ and cognitive change were made for both Whites and African Americans. Our study covered an extended period of time for the cognitive change. We used composite cognitive scores based on our previous factor analysis, thus less likely to be limited by the floor or ceiling effects seen in many individual tests. Consensus diagnosis of dementia and MCI was determined according to standard research criteria. Finally, measures for multiple potential confounding factors have been carefully recorded and adjusted in the analyses.

Taken together, our results suggest that positive or greater burden of Aβ in the brain is associated with accelerated decline in executive/speed function in the years prior to the PET scanning. In addition, our findings suggest further investigation of the implication of PET Aβ deposition on cognition, while taking into account factors such as ethnicity and APOE genotype.

## Supporting Information

S1 File Supplementary tables. (DOCX)
