# Supplementary material for: Brain Amyloid Deposition and Longitudinal Cognitive Decline in Nondemented Older Subjects: Results from a Multi-Ethnic Population
Source: PLoS One. 2015 Jul 29;10(7):e0123743. doi: 10.1371/journal.pone.0123743 (PMC4519341; doi:10.1371/journal.pone.0123743)
Supplement: S1 File — (DOCX) [file pone.0123743.s001.docx]

# **Table A. Cognitive score and brain Aβ burdens by APOE genotype and gender.**

|  | No ɛ4 | 1 ɛ4 allele | 2 ɛ4 alleles | p | Males | Females | p |
| --- | --- | --- | --- | --- | --- | --- | --- |
| Number of subjects | 79 | 33 | 4 |  | 42 | 74 |  |
| Mean Cognition Z-score, mean (SD) | 0.36 (0.49) | 0.45 (0.47) | 0.30 (0.33) | 0.63 | 0.33 (0.47) | 0.41 (0.49) | 0.38 |
| Memory Z-score, mean (SD) | 0.18 (0.67) | 0.26 (0.7) | -0.24 (0.81) | 0.38 | 0.08 (0.68) | 0.26 (0.68) | 0.20 |
| Language Z-score, mean (SD) | 0.49 (0.56) | 0.62 (0.54) | 0.44 (0.4) | 0.49 | 0.56 (0.47) | 0.51 (0.59) | 0.60 |
| Visuospatial Z-score, mean (SD) | 0.42 (0.47) | 0.48 (0.39) | 0.57 (0.23) | 0.69 | 0.47 (0.45) | 0.43 (0.43) | 0.63 |
| Speed Z-score, mean (SD) | 0.4 (0.87) | 0.43 (0.79) | 0.42 (0.43) | 0.97 | 0.20 (0.89) | 0.53 (0.77) | 0.05 |
| Global SUVR, mean (SD) | **1.20 (0.17)** | **1.43 (0.25)** | **1.29 (0.24)** | **<0.0001** | **1.21 (0.19)** | **1.3 (0.23)** | **0.03** |
| FRC SUVR, mean (SD) | **1.18 (0.19)** | **1.41 (0.27)** | **1.31 (0.25)** | **<0.0001** | 1.13 (0.18) | 1.23 (0.22) | 0.07 |
| TMP SUVR, mean (SD) | **1.13 (0.16)** | **1.33 (0.26)** | **1.18 (0.24)** | **<0.0001** | **1.19 (0.21)** | **1.28 (0.25)** | **0.02** |
| PAR SUVR, mean (SD) | **1.12 (0.19)** | **1.33 (0.26)** | **1.19 (0.24)** | **<0.0001** | **1.11 (0.2)** | **1.22 (0.24)** | **0.03** |
| CG SUVR, mean (SD) | **1.38 (0.19)** | **1.64 (0.25)** | **1.46 (0.25)** | **<0.0001** | 1.39 (0.22) | 1.49 (0.25) | 0.05 |

# **Table B. Characteristics of study participants according to negative or positive visual reading of brain Aβ imaging.**

#

|  | Males | | | |  | Females | | | |
| --- | --- | --- | --- | --- | --- | --- | --- | --- | --- |
|  | Total | Negative | Positive | *p* |  | Total | Negative | Positive | *p* |
| Number of subjects | 42 | 30 | 12 | / |  | 74 | 45 | 29 | / |
| Follow up time(years), mean (SD) | 11.5 (2.6) | 11.2 (2.4) | 12.2 (2.9) | 0.22 |  | 12.1 (3.0) | 11.9 (3.1) | 12.2 (3.0) | 0.72 |
| Age (years), mean (SD) | 83.65 (4.2) | 82.97 (3.7) | 85.37 (5.1) | 0.15 |  | 85.0 (4.7) | 84.3 (4.7) | 86.1 (4.6) | 0.12 |
| Education (years), mean (SD) | 13.3 (3.8) | 13.8 (3.7) | 12.2 (3.9) | 0.21 |  | 12.1 (3.1) | 12.1 (4.3) | 12.8 (3.4) | 0.45 |
| Race/Ethnicity, N(%) |  |  |  | 0.92 |  |  |  |  | 0.38 |
| White | 20 (47.6) | 15 (50) | 5 (42) |  |  | 20 (27.0) | 9 (20.0) | 11 (37.9) |  |
| African-Americans | 17 (40.5) | 12 (40) | 5 (42) |  |  | 36 (48.6) | 24 (53.3) | 12 (41.4) |  |
| Hispanic | 5 (11.9) | 3 (10) | 2 (17) |  |  | 17 (23.0) | 12 (26.7) | 5 (17.2) |  |
| Other |  |  |  |  |  | 1 (1.4) |  | 1 (3.4) |  |
| APOE ε4 status, N(%) |  |  |  | 0.24 |  |  |  |  | **0.021** |
| 0 ε4 allele | 30 (71) | 23 (77) | 7 (58) |  |  | **48 (67)** | **34 (79)** | **14 (48)** |  |
| 1 ε4 allele | 12 (29) | 7 (23) | 5 (42) |  |  | **20 (28)** | **7 (16)** | **13 (45)** |  |
| 2 ε4 alleles | / | / | / |  |  | **4 (5.6)** | **2 (4.7)** | **2 (6.9)** |  |
| APOE ε4+, N(%) | 12 (28.6) | 7 (23.3) | 5 (41.7) | 0.24 |  | **23 (32.9)** | **9 (20.9)** | **14 (51.9)** | **0.01** |
| MCI, N(%) | 8 (19) | 4 (13.3) | 4 (33.3) | 0.14 |  | 9 (12.9) | 6 (14.0) | 3 (12.9) | 0.73 |
| Mean Cognition Z-score, mean (SD) | 0.33 (0.47) | 0.39 (0.49) | 0.19(0.37) | 0.21 |  | 0.41 (0.49) | 0.41 (0.54) | 0.41 (0.40) | 0.99 |
| Memory Z-score, mean (SD) | 0.08 (0.68) | 0.20 (0.61) | -0.21 (0.77) | 0.08 |  | 0.26 (0.68) | 0.32 (0.72) | 0.15 (0.63) | 0.31 |
| Language Z-score, mean (SD) | 0.56 (0.47) | 0.63 (0.47) | 0.40 (0.47) | 0.16 |  | 0.51 (0.59) | 0.44 (0.62) | 0.61 (0.53) | 0.24 |
| Visuospatial Z-score, mean (SD) | 0.47 (0.45) | 0.49 (0.51) | 0.44 (0.28) | 0.75 |  | 0.43 (0.43) | 0.38 (0.49) | 0.52 (0.30) | 0.18 |
| Speed Z-score, mean (SD) | 0.20 (0.89) | 0.23 (0.94) | 0.13 (0.77) | 0.76 |  | 0.53 (0.77) | 0.61 (0.79) | 0.40 (0.73) | 0.26 |
| Number of subjects with SUVR | 38 | 29 | 9 |  |  | 67 | 43 | 24 |  |
| **Global SUVR^┼^, mean (SD)** | **1.21 (0.19)** | **1.14 (0.15)** | **1.43 (0.15)** | **<0.0001** |  | **1.30 (0.23)** | **1.19 (0.13)** | **1.5 (0.25)** | **<0.0001** |
| **FRC SUVR^┼^, mean (SD)** | **1.19 (0.21)** | **1.11 (0.16)** | **1.43 (0.16)** | **<0.0001** |  | **1.28 (0.25)** | **1.16 (0.14)** | **1.49 (0.27)** | **<0.0001** |
| **TMP SUVR^┼^, mean (SD)** | **1.13 (0.18)** | **1.08 (0.14)** | **1.28 (0.2)** | **0.002** |  | **1.23 (0.22)** | **1.12 (0.11)** | **1.42 (0.24)** | **<0.0001** |
| **PAR SUVR^┼^, mean (SD)** | **1.11 (0.20)** | **1.04 (0.15)** | **1.34 (0.17)** | **<0.0001** |  | **1.22 (0.24)** | **1.11 (0.13)** | **1.41 (0.26)** | **<0.0001** |
| **CG SUVR^┼^, mean (SD)** | **1.39 (0.22)** | **1.31 (0.17)** | **1.66 (0.14)** | **<0.0001** |  | **1.49 (0.25)** | **1.38 (0.16)** | **1.68 (0.27)** | **<0.0001** |

# **Table C. Cross-sectional association between Aβ level and cognition among MCI and cognitively healthy subjects.**

|  | Global | | FRC | | TMP | | PAR | | CG | |
| --- | --- | --- | --- | --- | --- | --- | --- | --- | --- | --- |
|  | B | *p* | B | *p* | B | *p* | B | *p* | B | *p* |
| Mean | 0.02 | *0.83* | -0.08 | *0.36* | 0.12 | *0.20* | 0.02 | *0.80* | 0.03 | *0.75* |
| Memory | 0.21 | *0.57* | 0.13 | *0.70* | 0.21 | *0.56* | 0.19 | *0.58* | 0.24 | *0.49* |
| Language | 0.25 | *0.32* | 0.19 | *0.42* | 0.31 | *0.22* | 0.23 | *0.33* | 0.19 | *0.43* |
| Visuospatial | 0.03 | *0.71* | -0.10 | *0.20* | 0.12 | *0.17* | 0.02 | *0.79* | 0.10 | *0.23* |
| Speed | -0.33 | *0.08* | -0.32 | *0.06* | -0.27 | *0.15* | -0.28 | *0.10* | -0.31 | *0.08* |

Results from Generalized linear models, adjusted for age at PET scan, sex, education, ethnic groups, and APOE ε4 genotype.

# **Table D. Cognitive scores and Aβ level among MCI and cognitively healthy subjects.**

|  | All subjects | Non-MCI | MCI | p |
| --- | --- | --- | --- | --- |
| N | 116 | 99 | 17 | / |
| Follow up time(years), mean (SD) | 11.84 (2.9) | 11.79 (2.85) | 12.10 (3.25) | 0.69 |
| Age (years), mean (SD) | 84.51 (4.58) | 84.53 (4.55) | 84.38 (4.92) | 0.90 |
| Education (years), mean (SD) | 12.71 (3.92) | 12.62 (3.86) | 13.24 (4.35) | 0.55 |
| Race/Ethnicity, N(%) |  |  |  | 0.12 |
| White | 40 (35) | 31 (31) | 9 (53) | / |
| African-Americans | 53 (46) | 45 (46) | 8 (47) | 0.93 (vs. Whites) |
| **Hispanic** | **22 (19)** | **22 (22)** | **0 (0)** | **0.02 (vs. Whites)** |
| Other | 1 (0.9) | 1 (1) | 0 (0) | / |
| APOE ε4+, N(%) | 35 (31) | 30 (32) | 5 (29) | 0.86 |
| Positive Clinical Reading, N(%) | 41 (35) | 34 (34) | 7 (41) | 0.59 |
| Female, N(%) | 74 (64) | 65 (66) | 8 (53) | 0.31 |
| Cognitive score at the first visit |  |  |  |  |
| **Mean Cognition Z-score, mean (SD)** | **0.64 (0.43)** | **0.68 (0.44)** | **0.45 (0.34)** | **0.05** |
| **Memory Z-score, mean (SD)** | **0.63 (0.66)** | **0.68 (0.65)** | **0.34 (0.64)** | **0.05** |
| **Language Z-score, mean (SD)** | **0.6 (0.53)** | **0.65 (0.51)** | **0.31 (0.58)** | **0.014** |
| Visuospatial Z-score, mean (SD) | 0.58 (0.43) | 0.59 (0.43) | 0.47 (0.43) | 0.28 |
| Speed Z-score, mean (SD) | 0.77 (0.55) | 0.78 (0.57) | 0.68 (0.41) | 0.47 |
| Cognitive score at the scan visit |  |  |  |  |
| **Mean Cognition Z-score, mean (SD)** | **0.38 (0.48)** | **0.44 (0.47)** | **0.03 (0.43)** | **<0.0001** |
| **Memory Z-score, mean (SD)** | **0.19 (0.68)** | **0.29 (0.66)** | **-0.40 (0.55)** | **<0.0001** |
| **Language Z-score, mean (SD)** | **0.53 (0.55)** | **0.59 (0.51)** | **0.17 (0.63)** | **0.003** |
| Visuospatial Z-score, mean (SD) | 0.45 (0.44) | 0.47 (0.43) | 0.30 (0.44) | 0.15 |
| Speed Z-score, mean (SD) | 0.41 (0.83) | 0.45 (0.8) | 0.13 (0.99) | 0.18 |
| Number of subjects with SUVR | 105 | 89 | 16 |  |
| Global SUVR, mean (SD) | 1.27 (0.22) | 1.27 (0.23) | 1.23 (0.19) | 0.44 |
| FRC SUVR, mean (SD) | 1.25 (0.24) | 1.25 (0.25) | 1.22 (0.21) | 0.59 |
| TMP SUVR, mean (SD) | 1.19 (0.21) | 1.20 (0.22) | 1.14 (0.16) | 0.34 |
| PAR SUVR, mean (SD) | 1.18 (0.23) | 1.19 (0.23) | 1.12 (0.19) | 0.28 |
| CG SUVR, mean (SD) | 1.45 (0.24) | 1.46 (0.25) | 1.43 (0.20) | 0.64 |
